# Fruit and vegetable intake among psychiatric inpatients: an electronic health record-based study

**DOI:** 10.1192/bjo.2021.944

**Published:** 2021-06-18

**Authors:** Adelaide Yue, Aida Seyedsalehi, Jonathan Lewis, Konstantinos Ioannidis, Julia Deakin

**Affiliations:** 1 School of Clinical Medicine, University of Cambridge; 2 Department of Public Health and Primary Care, University of Cambridge, Cambridge and Peterborough NHS Foundation Trust; 3 Cambridge and Peterborough NHS Foundation Trust; 4 Cambridge and Peterborough NHS Foundation Trust, Department of Psychiatry, University of Cambridge, Faculty of Health Medicine and Life Sciences, University of Maastricht

## Abstract

**Aims:**

Psychiatric illness is associated with premature mortality, which is largely attributable to physical health conditions. Low fruit and vegetable intake is a risk factor for cardiovascular disease, which contributes significantly to this disparity in physical health. This study used routinely collected data from electronic health records to assess fruit and vegetable intake among psychiatric inpatients across a UK mental health trust.

**Method:**

We conducted an anonymised search of de-identified electronic patient records from the Cambridgeshire and Peterborough NHS Foundation Trust (CPFT) research database. We collected data on ICD-10 diagnosis and fruit and vegetable intake for patients aged 18 years or over, with a recorded ICD-10 psychiatric diagnosis, admitted to CPFT inpatient facilities between March 2013 and January 2019 inclusive (n = 1031). Information on fruit and vegetable intake is recorded as part of a General Health and Lifestyle questionnaire, routinely performed within a week of admission. Fruit and vegetable intake in different ICD-10 diagnostic categories was compared using a one-way ANOVA.

**Result:**

Among patients for whom data on fruit and vegetable intake was recorded (n = 768), the prevalence of low fruit and vegetable intake (defined as <5 portions/day) was 75.9%, and mean fruit and vegetable intake was 2.85 portions/day (95% CI 2.72-2.98). Fruit and vegetable intake was lowest among patients with schizophrenia (mean = 2.3 portions/day), significantly worse than other diagnostic groups. In patients with schizophrenia, prevalence of low fruit and vegetable intake was 86.5%.

**Conclusion:**

Low fruit and vegetable intake is common among CPFT psychiatric inpatients, particularly those with schizophrenia. Interventions to improve dietary habits, such as increasing tailored for individuals with psychiatric illness may help to reduce the risk of physical illness.

